# Three Dimensional-Printed Gingivectomy and Tooth Reduction Guides Prior Ceramic Restorations: A Case Report

**DOI:** 10.3390/dj12080245

**Published:** 2024-08-01

**Authors:** Carlos A. Jurado, Jose Villalobos-Tinoco, Mark A. Lackey, Silvia Rojas-Rueda, Manuel Robles, Akimasa Tsujimoto

**Affiliations:** 1 Division of Operative Dentistry, Department of General Dentistry, Health Science Center, College of Dentistry, The University of Tennessee, Memphis, TN 38163, USA; 2 Department of Restorative Dentistry, Centro de Estudios Odontologicos (CEO), Queretaro 76050, Mexico; 3 Private Practice and Independent Researcher, Culiacan 80030, Mexico; 4 Department of General Dentistry, Health Science Center, College of Dentistry, The University of Tennessee, Memphis, TN 38163, USA; 5 Division of Dental Biomaterials, Department of Clinical and Community Sciences School of Dentistry, The University of Alabama at Birmingham, Birmingham, AL 35233, USA; 6 Department of Prosthodontics, Universidad del Valle de Mexico, Hermosillo 83165, Mexico; 7 Department of Operative Dentistry, School of Dentistry, Aichi Gakuin University, Nagoya 464-8651, Japan; 8 Department of Operative Dentistry, College of Dentistry, University of Iowa, Iowa City, IA 52242, USA; 9 Department of General Dentistry, School of Dentistry, Creighton University, Omaha, NE 68102, USA

**Keywords:** 3D printing, additive technology, gingivectomy, tooth preparations, CAD/CAM technology, ceramic veneers

## Abstract

Computer-aided design and computer-aided manufacturing (CAD/CAM) dentistry have significantly changed workflows in recent years. Restorations and devices can now be digitally designed and 3D-printed for dental care purposes. This clinical case report provides straightforward protocols for the digital design and 3D manufacture of gingivectomy and tooth preparation guides. These types of guides improved the gingival architecture of the anterior teeth and provided controllable tooth preparations prior to labial ceramic veneers. Thoughtful clinical evaluation started with listening to the patient’s chief complaint and extra- and intra-oral evaluations. Then a digital wax-up was performed, followed by an intra-oral mock-up, to evaluate the shape of the proposed restorations. After patient acceptance, the clinical procedure started with the gingivectomy and tooth preparation. Hand-crafted porcelain veneers were bonded under rubber dam isolation to avoid any contamination and maximize the bonding protocol. The esthetic and functional demands were fully satisfied. Predictable outcomes can be obtained whenever a meticulous evaluation and execution of all the steps are performed. Three dimensional printing technology allows the fabrication of devices such as gingivectomy and tooth reduction guides that help accomplish the desired results.

## 1. Introduction

Demand from patients seeking esthetic dental care has been rising over the last few years [1]. However, esthetic dental care not only deals with the shade and shape of anterior teeth but also with improving the functional and biological conditions, and it may require a multidisciplinary approach including restorative, periodontal, orthodontic, and surgical approaches [2]. Esthetic restorative dental treatments using ceramic veneers have been shown to boost the self-confidence of patients [3]. Furthermore, social media has also increased the demand for dental esthetic treatments. A recent cross-sectional study surveyed dentists regarding dental esthetic procedures among social media users, and the results indicated that 54.7% of the procedures were teeth whitening, 17% were Hollywood smile makeovers, 11.9% were dental veneers, and 10.4% were Invisalign procedures. The authors concluded that there is a shift in patients’ self-image toward seeking the perfect smile [4].

Novel technology has significantly improved dental workflows, and it allows clinicians to digitally design and evaluate the proposed dental care prior to any irreversible treatment [5,6]. Digital workflows have been shown to expedite the fabrication of the final prostheses in comparison to traditional methods [7,8]. Patients have also been shown to be in favor of the utilization of innovative technology for their dental care, and a recent systematic review and meta-analysis evaluating patient-related outcomes of conventional impressions versus intraoral scanning for prosthetic rehabilitations was conducted. The results showed that traditional impressions have higher discomfort, nausea, unpleasant taste, and breathing difficulty, and the authors concluded that intraoral scanners offer a suitable alternative to conventional impressions, offering a better patient’s experience [9].

Additive technology is one of the newest implementations in CAD/CAM technology, and it offers the fabrication of interim and final prostheses and different devices needed for dental care [10]. The fabrication of 3D models has been shown to be clinically acceptable in dentistry. A recent study evaluated the implant prosthesis accuracy and fit of 3D-printed and conventional stone models. The study evaluated the fit of two internal connection implants in the anterior maxilla; 10 conventional open tray impressions were made to create the stone models; and 10 digital scans were taken to print modes with four different 3D printers. The results displayed an error of 94.6 μm for the stone cast group and 46.9 μm for the 3D-printed models. The authors concluded that 3D-printed models demonstrated lower deviations than stone casts [11]. Furthermore, a recent systematic review evaluated novel versus traditional workflows for the production of physical casts for fixed prosthodontic treatments. The review initially assessed 613 publications, and it concluded that both 3D printing technology and traditionally manufactured casts offer similar precision within an acceptable range [12].

Tooth reduction guides have been recommended in order to obtain a more controlled removal of tooth structure. Traditional techniques employ reduction guides made of putty [13,14], thermoplastic sheet [15], resin composite [16], or metal [17]. Moreover, it has been demonstrated that ideal and uniform preparations provide optimum periodontal health, structural durability, and aid in obtaining high esthetic results [18,19,20]. On the contrary, freehand tooth preparations can also be performed for veneer restorations; however, this may represent a challenge for inexperienced clinicians. A recent study compared the amount of tooth removal for veneer preparations with free-hand, silicone guides, thermoplastic sheets, and two different printed reduction guides, and the results indicated that the least accurate preparation was the free-hand technique [21]. Therefore, it is recommended to use some type of reduction guide in order to assure the minimum required tooth removal for veneer restorations.

Recent clinical reports in the literature have shown novel techniques to digitally design and 3D print tooth reduction guides [22,23]. This type of novel printed reduction guide allows tooth preparation on different surfaces, such as the facial, incisal, and interproximal, in order to offer a more precise contour. However, no report has combined 3D-printed guides to perform gingivectomy and tooth preparation on the same patient. Therefore, the aim of this case report is to describe a young female patient seeking to improve her smile because she noticed space between teeth and stained previous composite restorations between the central incisors. The patient was offered ceramic laminate veneers and gingivectomy procedures performed with digitally designed and 3D-printed guides for gingivectomy and minimally invasive tooth preparations for hand-crafted ceramic restorations.

## 2. Materials and Methods

**Figure 1:**
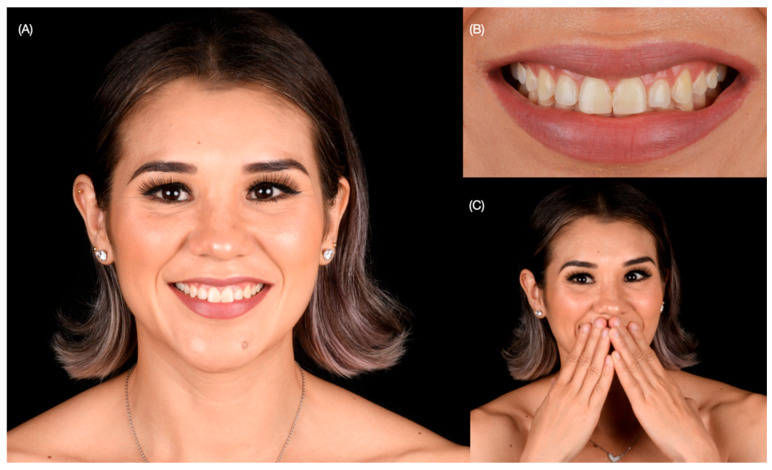
Initial extra-oral situation. (**A**) Face smiling, (**B**) close-up of the smile, and (**C**) patient disliking to smile.

**Figure 2:**
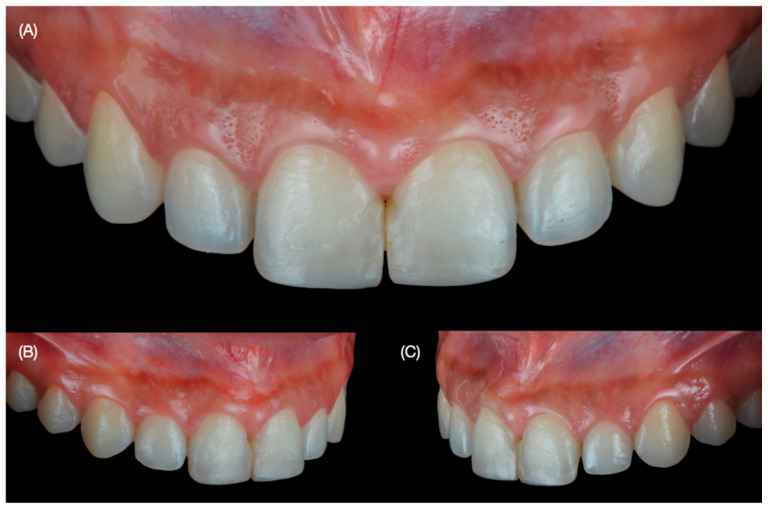
Initial intra-oral situation. (**A**) Frontal view, (**B**) right side view and (**C**) left side view.

A 30-year-old female patient presented to the university clinics with the main complaint of improving her smile. The patient dislikes smiling, and she wants to close the spaces between teeth and improve the size of some teeth (Figure 1 and Figure 2).

After clinical assessment, she was diagnosed with spaces between 21 and 23; uneven incisal edges of 11 and 21; and a non-ideal shape of tooth 13. The patient had orthodontic treatment 10 years before, and part of the resin used to bond the brackets remained on the facial surface of the anterior teeth. Moreover, old and stained composite restorations between the central incisors are present. The patient was offered to combine orthodontic treatment to close spaces, followed by tooth whitening and minimally invasive veneer restorations. However, she rejected orthodontic treatment since she was dissatisfied with previous treatments, wanted to avoid tooth whitening procedures, and only requested ceramic veneers. She was informed of the need to have a diagnostic wax-up and an intra-oral mock-up to evaluate the proposed restorations.

**Figure 3:**
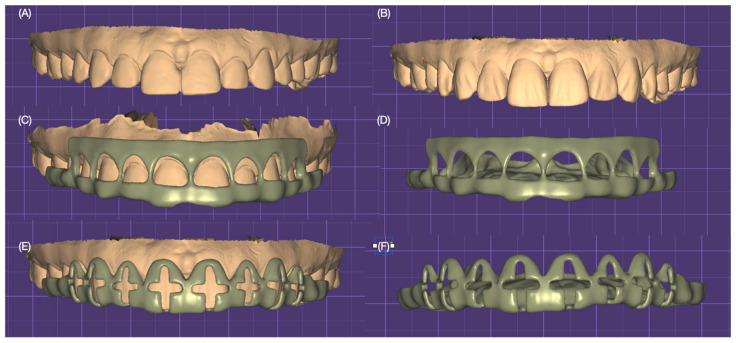
Digital design of the 3D-printed gingivectomy and tooth reduction guide; (**A**) initial situation; (**B**) digital wax-up; (**C**) gingivectomy guide design over the digital wax-up; (**D**) gingivectomy guide alone frontal view; (**E**) cross-shaped tooth reduction guide over the digital wax-up; and (**F**) cross-shaped guide alone frontal view.

Diagnostic impressions were made with polyvinyl siloxane material (Virtual 380, Ivoclar Group, Schaan, Liechtenstein) and poured out with type IV stone (Fujirock, GC, Tokyo, Japan), followed by a facebow record and mounted on a semi-adjustable articulator (Artex CR, Amann Girrbach, Vorarlberg, Austria). Diagnostic casts were scanned with a desktop scanner (Degree of Freedom HD, DOF, Seoul, Republic of Korea). A digital wax-up with the desired tooth contours was performed, followed by the design of gingivectomy for improving the gingival architecture for both lateral incisors and tooth reduction guides (Figure 3).

**Figure 4:**
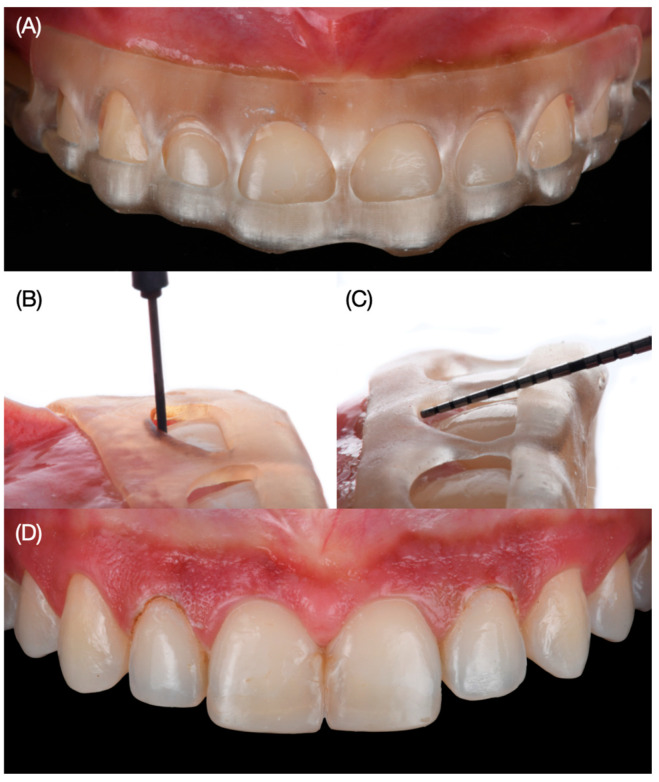
Gingivectomy procedure. (**A**) Placement of the printed guide, (**B**) gingivectomy procedure, (**C**) measuring with periodontal probe, and (**D**) result of the gingivectomy frontal view.

Digital wax-up models were printed out (Anycubic Resin 3D Printer Mono 4K, Anycubic, Shenzhen, China) of model resin and putty guides (Elite P&P, Zhermack, Badia Polesine, Italy). Due to the patient’s wide smile, clinicians proposed veneer restoration from the right second premolar to the left second premolar. An intra-oral mock-up was performed with a self-cured interim composite material (Structure Premium, VOCO GmbH, Cuxhaven, Germany) following the proposed shade of the restorations and gingival architecture, and the patient approved the proposed restorations and the new position of the zeniths. Gingivectomy and tooth reduction guides were printed with clear resin (Anycubic Clear UV Resin, Anycubic, Shenzhen, China). Even though the gingivectomy procedure was only planned for both lateral incisors, the facial windows following the ideal gingival zenith position were also created for all anterior teeth in order to verify clinically the gingival situation so there could be a comparison between the digital plan and the actual physical position of the soft tissue. At the following appointment, the gingivectomy guide was placed, and the procedure was performed with an electrosurgical unit (Sensimatic 700SE Electrosurge, Parkell, Edgewood, NY, USA) (Figure 4).

**Figure 5:**
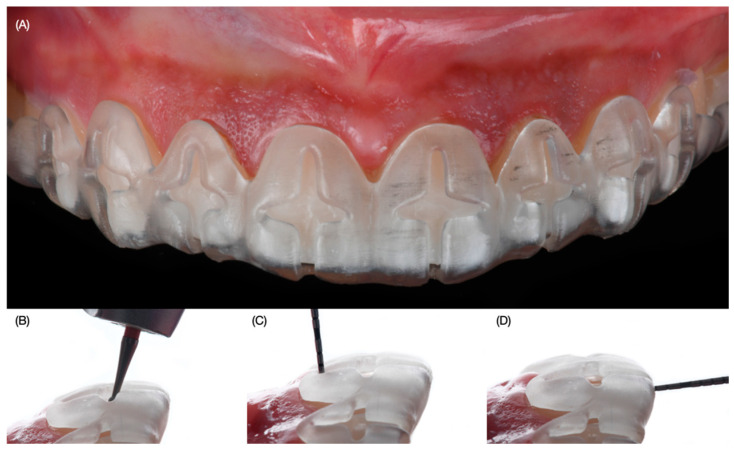
Tooth preparation with reduction guide. (**A**) Placement of the tooth reduction guide, (**B**) tooth preparation with the reduction guide in place, (**C**) measuring with periodontal probe at the gingival third and (**D**) at the incisal edge.

Then the diagnostic mock-up was placed intra-orally, a tooth reduction guide was placed over, and minimally invasive tooth preparations were created, initially with a fine diamond bur (801 Spherical, JOTA AG, Rüthi, Switzerland) to make horizontal and vertical grooves with 0.5 mm reduction and also 1.5 mm incisal reduction. The periodontal probe assisted with the measurement of the surfaces (Figure 5).

**Figure 6:**
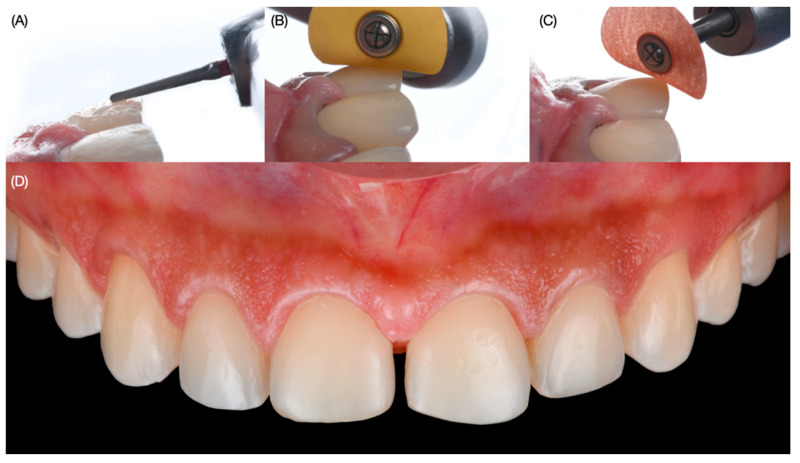
Finishing final tooth preparations. (**A**) Refining with fine diamond bur, (**B**) polishing with fine grift disc, (**C**) polishing with super fine grift disc, and (**D**) final polished preparations.

Then, the tooth reduction was taken on and off for the remaining tooth preparation with a specialized veneer kit (Efficient Veneer Prep Kit, JOTA AG, Rüthi, Switzerland) in order to closely monitor the reduction. Moreover, the periodontal probe also helped in the depth measurement of the preparation. Since the printed guide is clear, it allows for an overall evaluation of the underneath surfaces. The old and stained resin composite in between both central incisors was removed, and the final tooth preparations were polished with coarse, medium, and fine grit polishing discs (Sof-Lex XT Disc, 3M, St. Paul, MN, USA) (Figure 6).

**Figure 7:**
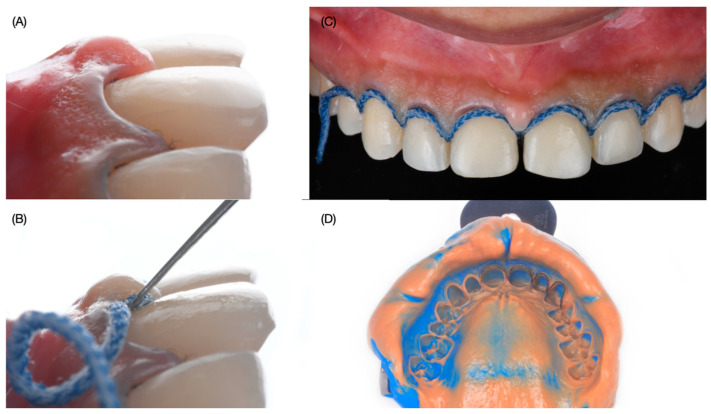
Final impression. (**A**) lateral view of final preps, (**B**) second cord packing process, (**C**) second cord packed, and (**D**) final impression.

Provisional veneer restorations were placed with a self-cured interim composite material (Structure Premium, VOCO GmbH, Cuxhaven, Germany). After two months of healing, the patient was ready to continue with the treatment. A double wet cord impression technique with #0 and #000 (Ultrapak, Ultradent, South Jordan, UT, USA) was made with polyvinyl siloxane material in heavy-body and light-body consistency (Virtual 380, Ivoclar Group, Schaan, Liechtenstein) (Figure 7).

**Figure 8:**
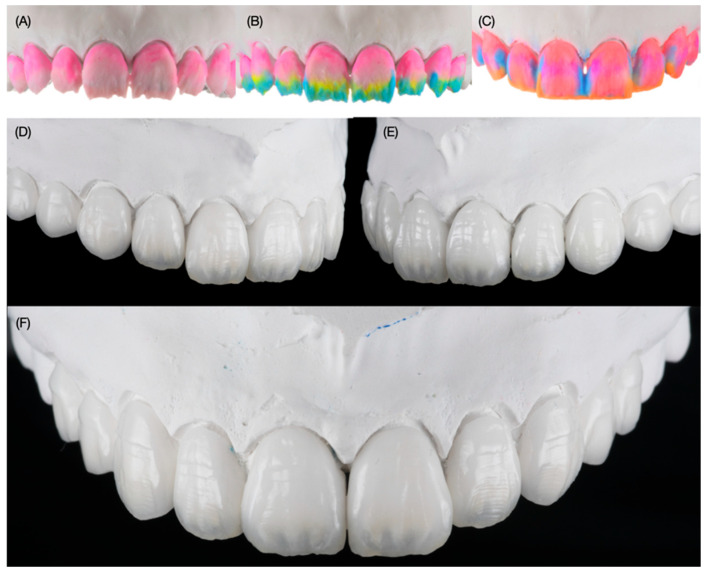
Fabrication of ceramic veneers. (**A**) Placement of body porcelain, (**B**) placement of translucent porcelain, (**C**) placement of porcelain with different shades, (**D**) final restorations right side view, (**E**) final restorations left side view, and (**F**) final restorations frontal view.

The final master cast was made out of type IV stone (Fujirock, GC, Tokyo, Japan), and the final restorations were hand-crafted with feldpsathic porcelain (Noritake Super Porcelain EX-3, Kuraray Dental, Tokyo, Japan) (Figure 8).

**Figure 9:**
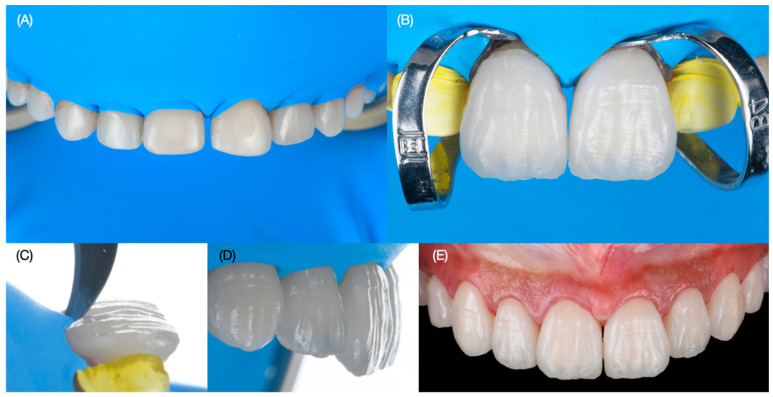
Bonding of the final restorations. (**A**) Rubber dam placement, (**B**) bonding of veneers for central incisors, (**C**) removing excess with blade, (**D**) final restoration’s lateral view, and (**E**) final restoration’s frontal view.

**Figure 10:**
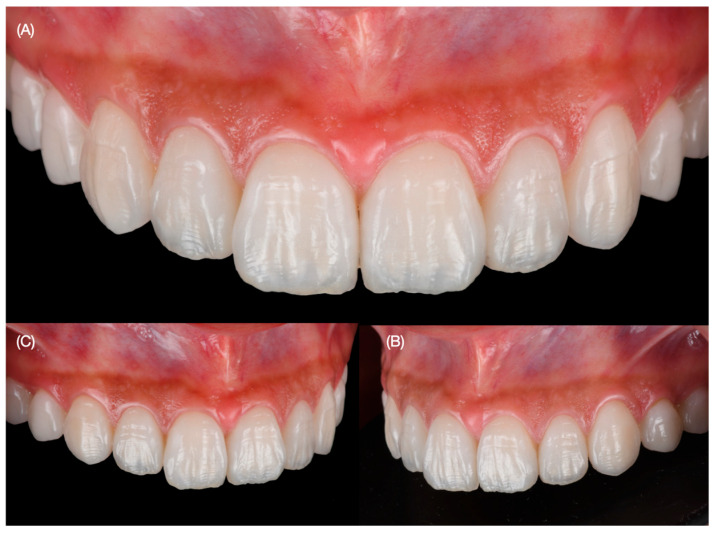
Intra-oral view of final restorations. (**A**) Frontal view (**B**) right side view, and (**C**) left side view.

The try-in of the veneers was accomplished to evaluate the fitting and try-in with light and neutral try-in pastes (Variolink Esthetic Try-in, Ivoclar Group, Schaan, Liechtenstein). The patient evaluated the results and selected a light shade, and she requested to continue with the cementation. Complete isolation was provided with a rubber dam (Nic Tone Dental Dam, MDC Dental, Guadalajara, Mexico) from the maxillary right first molar to the left first molar with holding clamps (Rubber Dam Clamps, no. 2, Hu-Friedy, Chicago, IL, USA) and single clamps (Hygenic Brinker Clamp B4, Coltene/Whaledent Inc., Cuyahoga Falls, OH, USA) placed on the specific teeth to which veneers would be bonded. The intaglio surface of the porcelain restorations was treated first with hydrofluoric acid (Ceramic Etching Gel, Ivoclar Group, Schaan, Liechtenstein) for 60 s, followed by rinsing and air drying, and then they were cleaned in an ultrasonic bath (5300 Sweep Ultrasonic Cleaner, Quala Dental Products, Nashville, TN, USA) with alcohol for five minutes. Then, silane coupling agent (Monobond S, Ivoclar Group. Schaan, Liechtenstein) was applied for 60 s and then restorations were dried. The surface of the teeth was treated first with water and 29-micron aluminum oxide particles (AquaCare Aluminum Oxide Air Abrasion Powder, Velopex, London, England) and then treated with 32% phosphoric acid (Uni-Etch w/BAC, Bisco Dental, Schaumburg, IL, USA) for 30 s and then rinsed and gently dried. Then primer and adhesive were applied (OptiBond FL, Kerr Dental, Brea, CA, USA) to the preparations and light cured (Valo Led, Ultradent, South Jordan, UT, USA) for 20 s, and finally light color resin cement (Variolink Esthetic LC, Ivoclar Group, Schaan, Liechtenstein) was applied to the veneers for both central incisors, and excess cement was removed with a microbrush and floss before applying curing light for 20 s on the facial, 20 s on the mesial, 20 s on the distal, and 20 s on the incisal surface. The same sequence was performed for the placement of the ceramic veneers for both lateral incisors, canines, and first and second premolars. Excess cement was eliminated with blade no. 12 (Surgical Scalpel Blade no. 12, Salvin Dental Specialties, Charlotte, NC, USA). Glycerin (Liquid Strip, Ivoclar Group, Schaan, Liechtenstein) was applied to the veneer restorations in order to prevent oxygen inhibition, and each surface was cured for 20 s. The rubber dam was removed, and occlusion, excursive movements, and protrusion were evaluated (Figure 9 and Figure 10).

**Figure 11:**
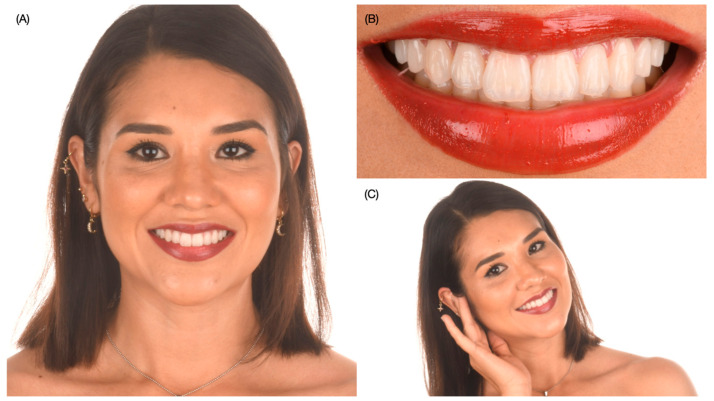
Extra-oral view of final restorations. (**A**) Full-face smile, (**B**) smile and (**C**) patient liking to smile.

The bonding procedure under dental isolation may take some time, and the clamps and dam create pressure on the tissues and teeth. Therefore, photos immediately taken after dental dam removal may display some gingival inflammation and small spaces between teeth, but after some time, the tissues return to their normal situation. The patient was satisfied with the shade, shape, and overall functional and esthetic outcome of the restorations (Figure 10 and Figure 11).

**Figure 12:**
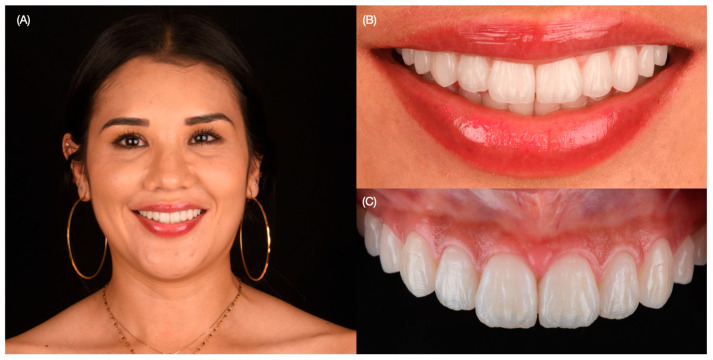
Three-year follow-up. (**A**) Face smiling, (**B**) smile, and (**C**) intra-oral frontal view.

A mouth guard was provided to wear at night in order to protect the ceramic veneers. At the three-year follow-up, she was still satisfied with the clinical result (Figure 12).

## 3. Results

**Figure 13:**
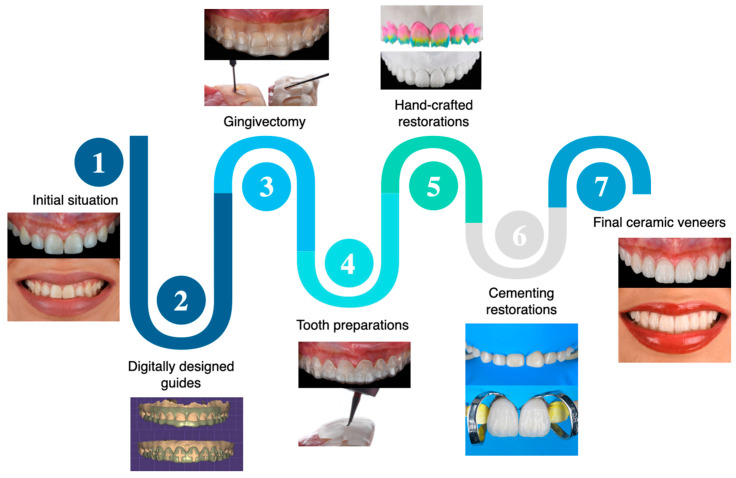
Flowchart describing the clinical workflow implemented for this dental treatment.

Digitally designed and 3D-printed gingivectomy and tooth reduction guides aid the clinician in improving the gingival architecture and performing minimally invasive tooth preparations in the esthetic zone. Thoughtful planning starts with dental photographs and evaluating the patient’s esthetic concerns. Dental photographs, digital diagnostic wax-up and design of the guides, printed reduction guides for gingivectomy procedures, minimally invasive tooth preparations, and bonded hand-crafted veneers under rubber dam isolation provided a predictable outcome that satisfied functional and esthetic demands. A flowchart describing the steps for clinical care can be seen in Figure 13.

## 4. Discussion

Esthetic dental care represents a challenging situation for clinicians because it requires thoughtful evaluation of the shade and shape of teeth, lip position, buccal corridors, incisal edge position, periodontal status, gingival zenith location, and patients’ expectations [24,25,26]. A photographic evaluation is recommended in order to document current dental conditions, educate the patient about proposed care, display the limitations, and communicate with a dental technician [27,28]. Clear communication with the patient regarding expectations is crucial in order to prevent misunderstandings, and this includes the number of appointments and procedures needed to achieve the desired outcome [29]. For the present report, the patient was very clear regarding her desire to improve her smile. Because the smile displayed non-ideal gingival architecture, a gingivectomy procedure was performed in order to improve the overall contours. Moreover, the patient has a wide smile displaying up to the second premolar, and therefore the minimally invasive veneer restorations were extended up to the second premolar.

A diagnostic wax-up is a fundamental step in the evaluation of the desired outcome [30]. This can be performed with or without modifying the cast. Additive diagnostic wax-ups do not require modification of the casts in order to perform them, but they do require the teeth to be aligned in an ideal position within the arch [31]. If the teeth are tilted, extruded, or in a non-ideal location, then the cast needs to be modified prior to the diagnostic wax-up [32]. Novel technology allows the fabrication of digital wax-up with and without modifying the teeth position. In the present case, our patient presented the teeth within the ideal arch location, and the digital wax-up performed only improved the proportion of the teeth.

Digital dentistry allows the planning of the dental procedure before any invasive treatment. The dual guide for gingivectomy and tooth reduction was digitally planned over the diagnostic wax-up. Three dimensional (3D)-printed reduction guides have been shown to offer a more controlled tooth reduction. A recent study evaluated the accuracy of the depth veneer preparations performed by freehand, silicone guide, thermoplastic guide, 3D-printed uniform, and 3D-printed with auto-stop. The guides were used to prepare a maxillary central incisor, and a digital scan was taken before and after the preparation in order to evaluate the amount of surface removed. The results of that study showed that freehand had the least accuracy for preparation depth with an error of 0.237 mm, followed by silicone guide (0.191 mm) and thermoplastic (0.149 mm), and the most accurate and conservative were 3D-printed uniforms (0.093 mm) and 3D-printed with auto-stop (0.059 mm). The authors concluded that the type of tooth reduction guide played a role in the accuracy of the preparation [33]. A recent systematic review evaluated the digital workflow of esthetic veneers from design to cementation. The study initially screened 158 manuscripts from different databases and evaluated the data regarding tooth preparation with reduction guides and freehand, and the authors concluded that tooth preparation guides provide a more accurate veneer preparation than freehand preparation [34]. For the present study, a digitally designed 3D-printed guide was used for the veneer preparation. Even though clinicians can perform the preparation without any guidance, it is highly suggested to utilize a type of preparation guide. The presented digital design was in a cross shape with an opening on the incisal edge. This type of design allows the clinician to perform the initial reduction on the facial and incisal surfaces, which are the most critical for veneer preparations. The gingivectomy guide was designed with the ideal zenith architecture of the anterior teeth, and the design included windows with a limit on zenith level in order to prevent excessive removal of soft tissue. Even though experienced surgeons may be able to perform gingivectomy procedures without any guide, it is recommended, especially for younger clinicians, to perform the procedure with a gingivectomy guide such as the present report in order to have more predictable results.

The final restorations were fabricated out of feldpsathic porcelain due to its highly esthetic properties and positive long-term data available. The literature has clinical reports in which feldspathic porcelain can be used to treat difficult clinical scenarios such as inherited enamel disorders such as amelogenesis imperfecta [35], enamel hypoplasia [36], and other complex situations such as closing diastemas and improving the color, shade, and position of teeth [37,38,39]. A clinical study evaluating the survival rate of feldspathic porcelain veneers evaluated 499 porcelain veneers placed on 155 patients by a single clinician. The tooth preparations had at least 80% enamel structure, and the results indicated a survival rate of 96% at 10 years and 91% at 20 years in service. The authors concluded that when bonded to an enamel substrate, the feldspathic porcelain veneers have excellent long-term survival with a low failure rate [40]. A recent systematic review evaluating the long-term and complication rates of porcelain laminate veneers assessed 2031 publications from different databases published in the last 25 years, and the results displayed a 95.5% survival rate of porcelain laminate veneers at 10 years [41].

The final restorations were bonded in total isolation with a rubber dam. The use of rubber dams provides several advantages to clinicians, such as preventing contamination, maximizing bonding properties, protecting patients by preventing swallowing or inhaling restorative instruments or materials, and improving the field of view [42,43,44]. Unfortunately, some clinicians dislike the placement of the rubber dam due to the time needed. However, in a survey of clinicians in Europe that assessed the time needed for rubber dam placement, 150 clinicians returned a survey with the information requested. The results indicated that the time needed for a rubber dam was 4 min for dental students and <2 min for dentists. The authors concluded that the time needed for rubber dam application was rather short [45]. Obviously, clinicians can perform bonding protocols without a rubber dam; however, it should be known that contamination of the field may compromise the bonding procedure and the longevity of the restoration.

The limitations of this clinical report are related to the use of only one type of tooth reduction guide, so future clinical scenarios should compare 3D-printed tooth reduction guides with different designs. Another limitation of this report is that it only provides a qualitative evaluation of the amount of tooth removal and does not provide a quantitative measurement of the tooth structure removal, so future reports should measure the amount of tooth preparation.

## 5. Conclusions

Novel 3D printing technology allows clinicians to fabricate devices that can expedite and facilitate dental care. Three dimensional (3D)-printed guides for gingivectomy and tooth preparations improved the gingival architecture of the anterior teeth and enabled minimally invasive preparations for labial ceramic veneers. This report describes an ideal clinical scenario for performing gingivectomy and veneer preparations with 3D printing technology.

## Data Availability

The original contributions presented in the study are included in the article, further inquiries can be directed to the corresponding author.
